# Exploring experiences of HIV care to optimize patient-centred care in Conakry, Guinea: a qualitative study

**DOI:** 10.3389/frph.2024.1134404

**Published:** 2024-04-09

**Authors:** Delphin Kolié, Etienne Guillard, Abdoulaye Sow, Hawa Manet, Bienvenu Salim Camara, Théophile Bigirimana, Mamane Harouna, Alexandre Delamou

**Affiliations:** ^1^ Ministry of Health and Public Hygiene, Centre National de Formation et de Recherche en Santé Rurale de Maferinyah, Forécariah, Guinea; ^2^ African Centre of Excellence for the Prevention and Control of Communicable Diseases, Gamal Abdel Nasser University of Conakry, Conakry, Guinea; ^3^ Solthis, Paris, France; ^4^ Fraternité Médicale Guinée, Conakry, Guinée; ^5^ Solthis, Conakry, Guinea

**Keywords:** HIV care, patient-provider relationship, qualitative research, psychosocial support, Guinea

## Abstract

**Introduction:**

Studies on the organisation of care and the power dynamic between providers and patients with HIV in sub-Saharan Africa are rare. This study aims to describe the patient-provider relationship and explore the challenges to optimal and patient-centred care for HIV patients.

**Methods:**

This was a qualitative exploratory descriptive study using in-depth individual interviews and focus group discussions. In total, 17 individual interviews and 5 focus group discussions were conducted. This was conducted in four urban health facilities in Conakry, the capital of Guinea. Three group of participants were included in this study namely patients with HIV; health providers including facilities and services managers; and psychosocial counsellors. Psychosocial counsellors provide emotional and psychosocial support to HIV patients. Their role in the organization care in Guinea is new and they contribute to strengthening adherence of patients with HIV to ARV treatment.

**Results:**

Patients with HIV, health providers, and psychosocial counsellors have a positive perception of the patient-provider relationship. This relationship was characterized essentially by maintaining confidentiality of HIV status disclosure, caring attitudes towards patients (being available, adjusting locations for accessing ART, based on patients’ preferences), and participating in HIV patient’s social life. However, scolding and miscommunication about the interpretation of viral load tests were reported. The shortage of human resources, low salaries of health staff, poor infrastructure, and the financial burden borne by patients with HIV impede the implementation of optimal patient-centred care.

**Conclusion:**

Integrating psychosocial counsellors in HIV care organization, improving access to ARV, infrastructure, increasing human resources, and removing the financial burden for HIV patients are needed to optimal patient-centred care in Guinea.

## Introduction

Sub-Saharan Africa has the highest burden of HIV infections (80%) (1). Accessing HIV care in this region remains a major concern for achieving the 90-90-90 UNAIDS targets (2). The UNAIDS 2014 report estimated that more than 50% of people living with HIV (PLHIV) in this region did not know their status and, for those who did, only 46% had access to antiretroviral treatment (ART), with 50% of PLHIV on treatment lacking access to viral load testing (1, 2).

Despite the overall decline in HIV incidence, mortality rate, and increase in treatment accessibility, several barriers (financial, geographical and acceptability of care) still impede the access to ART in sub-Saharan Africa (2, 3). The lack of infrastructure and human resources impact also the access to HIV care in this region (1, 2). Authors have found that the high stigma, low social support, and fear of judgment and unwanted status disclosure impede access to HIV care (4, 5). Alcohol abuse (for men) and the fear of domestic violence and abandonment (for women) have been reported to limit the disclosure of patients’ HIV status to their partners, as well as their access or adherence to care (4, 5). Associated non-HIV medication costs, transportation to and from health facilities have also been identified as barriers to HIV care (6). Poverty, unemployment and housing instability, and unfavourable clinic hours impede the sustained engagement of patients with HIV in care and treatment (7). Health system factors such as unfriendly provider, frequent stock-out of testing and drugs also influence the access and adherence to care of patients with HIV (8).

To overcome these challenges, there is a need to encourage HIV self-testing and to ensure there are adequate stocks of tests and drugs, affordable and acceptable health care services. Examples of these include engaging PLHIV in the design and delivery of ART services and enabling peer and lay providers to distribute ART refills and offer psychosocial support (2, 9). The development and implementation of patient-centred care might effectively contribute to enhancing HIV patients’ adherence to ART, their retention in care, and their viral load suppression (10).

A positive relationship with a health provider is perceived on the patient's side as the core determinant of success in adhering to long-term treatment (11). Studies have reported that there was increased adherence of HIV patients to ART if their provider cared about them (12, 13). A 2019 systematic review reported that discrimination of PLHIV reduced their motivation in initiating or continuing ART and exposed them to risky behaviour such as sexual promiscuity (14).

Therefore, it is essential from the outset to establish a patient-provider relationship of trust and mutual respect (13). Dang et al. identified five key factors that can reduce PLHIV anxiety and build a trust at the very early stage of the patient-provider relationship: (1) reassure patients (2) tell patients it is normal to ask questions (3) show patients their laboratory test results and explain what they mean (4) avoid stigmatizing language and behaviours (5) ask patients for treatment preference (13).

Yet there is limited evidence on the organization of HIV care in sub-Saharan Africa and on how different actors within health services perceive the patient-provider relationship. A study conducted in Kenya reported that the availability of ARTs and the reduction in patients’ waiting times resulted in increasing patients’ retention (11). Another study from Namibia found that patients who were empowered and trained were more likely to ask questions during consultations, enjoyed interactions with their providers and had good clinical outcomes (15). In such setting, organizations formed by people living with HIV can play a considerable role in supporting and empowering (15).

In Guinea, the prevalence of HIV/AIDS is 1.5% among adults in 2018 compared to 1.7% in 2008 (16, 17). Of the 98,481 people living with HIV, only 35.6% have access to anti-retroviral therapy (ART) (18). The challenges to HIV care in Guinea include low health service utilization, the poor quality of care received and the limited access to viral load tests (only 25% of current patients used ART testing in 2015) (18). This study aimed to examine the patient-provider relationship from the perspectives of different stakeholder groups—patients with HIV, health providers and managers, and members of organizations fromed by people living with HIV (psychosocial counsellors) in this study and explore the challenges to optimal and patient-centred care for PLHIV in Conakry, Guinea. Addressing such challenges would contribute to accelerating Guinea's path towards the attainment of international health objectives of ending HIV epidemic related public health threat by 2030. This study was undertaken to describe the patient-provider relationship and explore the challenges to optimal and patient-centred care for HIV patients in the context of the PACTES project implementation in HIV care facilities in Conakry.

### Study context: the justification of the PACTES project in Guinea

Guinea is located in West Africa with an estimated population of 10.5 million, living mainly in rural areas (70%) and below the poverty line (55%) (19).

The HIV epidemic is widespread in Guinea and is associated with a disparity between urban (2.7%) and rural (1.2%) settings (18). The city of Conakry remains the most affected setting (2.7%), compared to the national prevalence of 1.5% (18, 20). Moreover, nationwide, a large proportion of PLHIV (63%) do not know their status (21).

In 2016, the Ministry of Health implemented the “test all, treat all and retain all” policy (20). This is a policy which requires initiating ARTs for everybody tested HIV positive (20). Afterwards, a strategy was defined to integrate psychosocial counsellors in HIV care to facilitate the implementation of the “retain all” strategy. However, its implementation remains a challenge (20). About 80% of the funding for HIV/AIDS control activities depends on external sources (18, 19). Moreover, the shortages of skilled health workers constitute another challenge for the provision of quality healthcare services, including HIV care, in the country (22). According to the national census of 2014, Guinea accounted for nearly 9,222 skilled health workers; which corresponded to a ratio of 9 skilled health workers per 10,000 inhabitants (22). This ratio was approximately three times lower the threshold of 23 health workers per 10,000 inhabitants recommended by the world health organization in 2006 (22, 23). These health workers were also maldistributed in the country with 70% living in urban cities where 30% of the country population live (22). In the post-Ebola health system and workforce reforms, the government recruited 3,800 skilled health workers—including about 700 medical doctors, 1,000 nurses, 500 midwives, and 1,200 nurse assistants—in 2016 and deployed them in rural and remotes areas (24, 25).

A situation analysis of HIV care in Guinea conducted in 2016 (prior to the PACTES project) emphasised the low quality of HIV care including the limited coverage of the needs of patients with HIV (17, 26). Factors that underpinned this low quality of care included the inadequacy of care organisation to patients’ needs, the limited access to essential viral load tests, the lack of psychosocial support to patients, and the stigmatization and poor relationship between care providers and patients, including the poor listening skills of providers (17, 26). To address the challenges and contribute to the achievement of regional and international health objectives—end HIV epidemic related public health threat by 2030—the project “Patients au Cœur du Traitement et des Soins (patients at the forefront of treatment and care); PACTES” was initiated by the NGO SOLTHIS is collaboration with the Ministry of Health and Public Hygiene of Guinea (26). This project overall aimed to enhance quality of HIV care by testing new modes of patient-centred care including ensuring access to viral load testing, reorganizing care, training and supporting psychosocial counsellors to improve relationships between health providers and patients with HIV (26). Furthermore, this PACTES project also aimed to support organizations formed by people living with HIV to creating the necessary conditions for the emergence of strong community mobilisation in favour the access to better quality care (26). More specifically, the PACTES aimed to strengthen the capacity of five health facilities involved in HIV care provision in Conakry (four) and Kankan (one) provinces (26). Among other activities planned in this study sites between 2016 and 2019, included: empowering patients’ associations operating at health facility level (Psychosocial counsellors or mediators) and members of REGAP + and FEG associations (26). The Psychosocial counsellors are not part of the former health workforce involved in care provision in Guinea (17, 22). They were rather patients with HIV who were trained and financially motivated by the PACTES project (through their NGO/ associations) to provide peer education to other people with HIV including their adherence to follow-up visits and ART treatment on the project study sites (17). Specifically, they provide also pre and post-screening counselling to help people accept their status, adhere to treatment and provide therapeutic education (17). They also make home visits to people who are lost to follow-up (17). There were selected based on their experience with HIV associations, and their adherence to HIV care follow-up and ART treatment (26). In total, 50 Psychosocial counsellors were identified and trained in the project (26). They were also involved in the development process of tools to gather patients’ needs and expectations of HIV care (26). Furthermore, the project also targeted the training of Lab technicians across three identified laboratories in the intervention sites for viral load testing performing (26). The psychosocial support in the PACTES project therefore evolved an empowerment process of patients with HIV through their improved awareness about the right, roles and needs in the health care system (26).

Actors of the PACTES project assumed that Psychosocial counsellors’ involvement in the promotion of the right of patients with HIV and the experiences sharing with their peer could improve patients’ awareness about their roles, needs and expectations vis-à-vis the care circuit, and therefore empower patients with HIV in their care process. This in final would influence their approach of care and relationship with health providers.

## Methods

### Study design

We conducted a qualitative exploratory study (27). The study took place in Conakry, Guinea in 2018. We collected data in four public health facilities supported by a French -based non-governmental organization (NGO: SOLTHIS). These facilities stand for different levels of the health pyramid: two university hospitals, 1 primary health center and one associative health center.

### Study participants

The study population was composed of three groups of participants: (1) PLHIV, (2) health providers and managers and (3) psychosocial counsellors, (Tables 1, 2).

**Table 1:** Study participants’ characteristics.

Study participants	Socio demographic characteristics of study participants (IDI, FGD)
HIV patients	Men (45%) Women (55%), Youth/adults (average age 41.7 years old), unemployed as well as employed persons, such as veterinary surgeons/sellers/manual workers/drivers, people with or without formal education. Average number of children of the patients (2.44).
Psychosocial counsellors	Social workers, HIV patients, members of a local NGO promoting HIV patients’ rights.
Heath providers/managers	Contractors/volunteers/civil servants, health managers/physicians/laboratory technicians/nurses/pharmacists.

**Table 2:** Number of IDIs and FGDs per group of stakeholders and per site.

Study participants/ sites	1	2	3	4	Total
PLVIH patients	2 IDI	4 IDI	4 IDI	5 IDI	15 IDI
Psychosocial counsellors	1 FGD	–	–	–	1 FGD
Health providers / managers	2 IDI				2 IDI
	1 FGD	1 FGD	1 FGD	1 FDG	4 FGD
Total					**17 IDIs and 5 FGDs**

The bold values indicate the total number of individual interviews (IDIs) and focus group discussions (FGDs) conducted.

We used purposive sampling to recruit psychosocial counsellors and health managers (27). Psychosocial counsellors facilitated the identification and access to PLHIV. The criteria for internal diversification were gender, years of experience as health providers and HIV clinical care. A convenient sampling technique was used to recruit patients with HIV. In fact, the Psychosocial counsellors were asked to contact patients with HIV and inform them about the study. Only patients with HIV who accepted to participate in the study were contacted for interviews and included in this study. The Psychosocial counsellors were however asked to ensure a diversification of patients considering the following criteria: age, gender, employment status, and their adherence to ART treatment.

### Data collection

We conducted 17 in-depth interviews (IDI) with PLHIV, health providers, and managers. We conducted 15 IDIs (all were HIV-patients) in local languages (Sousou, Malinka, and Pular) and two in French, based on participants’ preferences (Table 2). The interviews were recorded for 16 participants. One participant refused to be recorded because of fear of confidentiality breach but systematic notes were taken.

We conducted interviews in private to ensure confidentiality. For two female patients, psychosocial counsellors attended the interviews at the patients’ request. An appropriate location for the interview was chosen for the four participants who asked not to be interviewed in the health facilities.

Additionally, we conducted five focus group discussions (FGDs) with health providers and psychosocial counsellors (Table 2). We recorded all the FGDs, each of which was composed of six to 10 participants.

On average, each IDI and FGD was of 45 min’ duration. For both data collection methods, we used pre-tested guides composed of four sections: socio-demographic characteristics, care pathway, actors’ perception of HIV care and patient-provider relationships. Only a few patients were interviewed in local language; all the other participants were interviewed in French, the official language of the country.

### Data analysis

We translated and transcribed all the IDIs and FDGs into French then analysed the transcripts using a thematic analysis approach (28). We used a mixed approach with deductive themes emerging from the research questions and inductive themes emerging from the empirical data (28). Software was not used to code the materials.

The diversity of data collection methods and data sources enabled us to triangulate the data and thus strengthened the internal validity of the study (29). To improve the consistency of the data and to avoid any potential bias, the principal investigator (DK) and three researchers (LB, HM and AD) conducted all individual interviews. In addition, content analysis of interviews was ensured by the two reviewers (DK and LB) using an Excel spreadsheet. Discrepancies were resolved by a team consultation that involved AD and BSC.

We shared and discussed the findings of the study with the SOLTHIS staff, then disseminated them to all the stakeholders (HIV patients, members of HIV associations, health providers and SOLTHIS project staff) during a one-day workshop. We validated the findings with the workshop participants.

### Ethics approval

The study protocol was approved by the National Ethics Committee for Health Research of Guinea (number: 077/CNERS/18). All study participants provided verbal informed consent before interviews.

### Patient and public involvement

Patient informed consent was obtained before conducting interviews.

## Results

### Role of psychosocial counsellors in HIV patients’ care

The quality of the multidisciplinary management team is an important element for ensuring effective adherence and continuity of care of HIV patients. Therefore, the importance of psychosocial counsellors has been accepted by all health system actors, including health providers.

Psychosocial counsellors, some of whom are peers, i.e., PLHIV involved in HIV care, were perceived as playing a role in reducing the health providers’ workload by dispensing educational therapy and psychosocial support to patients as well as filing records.

Some participants reported that psychosocial counsellors had a strong relationship with HIV patients attending health facilities. Their involvement in HIV care seemed to increase HIV patients’ confidence and improve their follow-up visits in health facilities. According to participants, the main enabler of this was the potential sharing of knowledge and experience with HIV patients which helped to prepare them to confront societal stigma. Some participants argued that the involvement of psychosocial counsellors had created a community of care where everyone talked about their difficulties and asked for advice.

*“…I was first diagnosed HIV positive during my antenatal visit… as I started taking medications and my child was born and confirmed HIV negative, I trusted the doctors’ [psychosocial counsellors] advice. I often come to meet them … …. (IDI#10, HIV patient, 35-year-old woman, widow)*.

*“We have no problem disclosing our status to HIV patients. This makes them more confident and not feel alone in their condition. You know, they sometimes hesitate because they feel they are different from others. We show them our medications, pictures of our children [HIV negative] and that makes them feel comfortable… (FGD#1, psychosocial counsellors)*

### A complex patient-provider relationship

Five themes emerged from the empirical data about how the patient**-**provider relationship in HIV care is perceived by the participants: confidentiality, caring-attitude, patients’ social life, scolding and miscommunication as well as decision-making.

#### Confidentiality in the process of care

Health providers emphasized the importance of confidentiality as the key dimension of patient-provider relationships that HIV patients valued the most. To ensure this confidentiality, health providers mentioned that the term ‘HIV’ was not written on office and examination room doors and only one HIV patient was received at a time.

*“…In the office, we make sure that the patient is not stigmatized… we do not let two patients enter the consultation room at the same time” (FGD#2, health providers)*.

Nevertheless, some patients reported that they usually avoided talking about their private life during the consultation with a health provider.

*"I do not tell them the problems of my family or my private life because you never know… you know here, people do not keep secrets” (IDI#6, HIV Patient, a 30-year-old woman, seller, divorced)*.

From the patients’ perspective, some said they had a ‘confident’ to whom they talk about their status. Others preferred not to reveal their status to anyone else because they fear confidentiality breach. Some others said they avoided attending the health facility in working hours as to avoid meeting a relative. Some would come at lunchtime or negotiate a weekend appointment while others would go to facilities located far from their homes.

*"I'm not stigmatized because nobody knows of my illness except my wife and I'm the only one who knows about hers too” (IDI#7, a 33-year-old male patient, married)*

#### Caring attitude

Patients reported that they were being cared for by their health providers and were provided with enough advice on how to behave to maintain a good state of health. However, this caring attitude was strongly focused on the drugs supply.

*“I was diagnosed in a hospital [health centre] located near [patient*’*s residence]. Since I came here, everyone has cared for me. as I live far away from here, they can provide me with 2–3 months’ of medication [ART drugs] (IDI#8, a 44-year-old female HIV patient widowed)*

Some patients appreciated that health providers were available for them and could help them with drug prescriptions.

*"The physicians here are fully available to us… any time you call them [over the phone], they are available to assist you… whenever I am sick, I call my physician and tell him that I want to take this or that product … “ (IDI#16, 46-year-old male HIV patient, married)*.

From the health provider's perspective, they feel the need to develop and maintain caring attitudes towards their patients. They stated that asking questions on what the patient did yesterday, their hobbies and food preferences could make the patient feel cared for. Furthermore, others reported that welcoming the patient warmly and comparing HIV to diabetes, or hypertension improved the patient-provider relationship.

*“ Everything depends on the way you welcome your patient … you have to ask what he does during his day, what he likes eating, did he go to a night club and what happened then… He [the patient] must be diverted from what he is suffering from.” (IDI#16, male health provider)*

#### Patients’ social life

Health providers reported that they contribute to improving the patients’ social life by assisting them in finding a partner.

*"There are patients who come to tell us that a friend (an HIV patient) wants to get married and is looking for a woman or a man to get in touch with. We manage to create families like that. And their care becomes very easy.” (FGD#4, health providers)*.

#### Paternalistic behaviour

Psychosocial counsellors and health providers reported that providers sometimes scolded at patients. However, they felt this could not be considered as misconduct but a way to make patients respect the care process.

*“The doctor shouts out because he [the patient] does not respect their appointments. This is for their well-being, not to harm the patient” (FGD#1 psychosocial counsellors)*.

*“We have a responsibility to tell them what will happen if they don*’*t follow our advice…. You have some patients who do not perform their tests at the scheduled period or who forget to take their medications sometimes. With such patients, you have to shout at them and make them obey the procedures … some patients are like that…” (IDI#17, health provider)*.

They also indicated poor communication between providers and patients, especially for laboratory results or viral load testing.

*“Saying to a patient that the results are good; what does good mean?” (FGD#1, psychosocial counsellors)*.

Some patients also mentioned that they were not aware about their viral load status although they had performed the test. This was reported to lead to miscommunication between patients and providers as some patients, according to participants, may not be aware of the implications of their treatment and the requirement to adhere to it.

#### HIV patients’ decision-making

HIV patients reported engaging in communication with their health providers, sharing their concerns and difficulties with them. Some emphasized that they could ask their providers to prescribe drugs according to their preferences. Others mentioned their ability to choose the health facility and the time they preferred for a medical visit.

Some health providers felt that respecting the patients’ choice of HIV care facilities was an appropriate way to retain them and get them to adhere to treatment.

*“He listens to me, advises me and encourages me … he prescribed me vitamins so I can eat and sleep well and gained weight before I go back to work” (IDI#7, 33- year-old male HIV patient, married)*

### Challenges to optimal and patient-centred care

#### Lack of human resources and motivation

At the health facility level, the shortage of human resources (both medical and psychosocial staff) was raised as an obstacle to implementing patient-centred care.

*"We only have two doctors here to look after all the patients”, (FGD#2, health providers)*.

The main concern raised by providers was their insecure status. Most of them were not civil servants and thus not paid regularly. This was seen as a demotivating factor that could influence the quality of a patient-provider relationship.

#### Poor infrastructure

Health providers and managers also mentioned the poor infrastructure that resulted in breaches in confidentiality as well as a lack of privacy during examinations.

*"We are four doctors consulting in this office here… We* only have one office for both reception and counselling of patients. *Our working conditions are really difficult here “(FGD#2, health providers)*.

#### Lack of drugs

During the data collection (July 2018), there was a serious ART stock-out following the ARV stockpile fire at Guinea's central pharmacy in June 2017. As a result, ARVs were being dispensed according to their availability, forcing patients to call or travel several times to the facilities to obtain drugs.

*"I came here and was told that there is no drug… I said how can we manage this because I live in Timi [fictitious name of a city located 50 km from Conakry]” (IDI#11, 29-year-old male HIV patient, single)*.

#### Financial burden for HIV patients

Although ARTs are dispensed free of charge, patients continued to pay for several services that can be very expensive, such as laboratory tests.

*“I paid 40,000 GNF [local currency, equivalent to 4USD] for transport as I live at Timi. I gave 300,000 GNF [30USD] to the Lab man and 20,000 GNF [2USD] to the Doctor who is taking care of me… I sold my phone for 100,000 GNF [10USD] and my shoes for 30,000 GNF [3 USD] (IDI#12 25-year-old male HIV patient, single, unemployed mechanic)*.

Furthermore, the cost of drugs to treat other diseases such as anaemia, diabetes or opportunistic diseases such as candidiasis or dermatitis is an additional financial burden for HIV patients:

*“HIV treatment is free but not the lab tests such as haemoglobin rate and glycaemia, nor the treatment of opportunist diseases. So, if a patient suffers from another disease, he has to pay for medications and related lab tests…” (FGD#2, health providers)*.

Patients also reported paying high transportation costs.

*“I usually come by motorcycle which costs 25 000 GNF (about 3 USD)"(IDI#12, a 34-year-old female HIV patient, unemployed, married)*.

Many participants reported that they preferred to use facilities a long distance from their place of residence to maintain the confidentiality of their status, even though it involved high transportation costs.

*“We have some patients who are registered in two or three different facilities. They can go to one facility for 3–6 months then change it to another… some even travel from one district to another to get their medications [ART drugs] (FGD#2, health providers)*.

## Discussion

This qualitative study provides an in-depth understanding of the organization of health care services and the patient-provider relationship in HIV care in Conakry, Guinea. Our findings show a less patriarchal patient-provider relationship, with a progressive engagement in their medical care on the part of HIV-patients’. Our study identified the role played by health providers in reconstructing patients’ social life and reorganizing the care pathway, taking in account the role of psychosocial counsellors. Study participants cited the financial burden associated with HIV care as an important hindrance, impeding patients’ adherence to care and reducing optimal patient-centred care.

### A patient-provider relationship focused on dispensing drugs

Studies in sub-Saharan Africa showed that HIV patients do not play an active role in their medical care. of the reasons include their low educational level, their experience of anxiety and fear of confidentiality breach or for being perceived as a difficult patient (13, 15, 30). In contrast, our findings identified changing behaviours and roles of both actors for building a confident relationship. HIV patients reported sharing their social and health concerns with health providers, communicating their drug prescriptions, and sometimes altering them according to their preferences. They also reported negotiating with health providers about their follow-up visits outside peak times and in their health facilities of choice.

On the health providers’ side, they reported using a variety of strategies to improve patients’ engagement in their care. As such, they reported building a caring and confident relationship with patients and reducing patients’ psychological burden by assimilating HIV to other diseases. However, our study identified drug provision and biomedical tests as essential components of the patient-provider relationship. From the patients’ perspective, the availability of ART was the central element in the improvement of their health. A reason might be the fact that HIV-patients in our study have good access to information on the efficacy of ART in improving their health and the consequences in interrupting ARTs. Ahmed S et al. found that HIV patients’ misconceptions of ARTs’ impact on their mortality and their preference for traditional medicine were among the factors that hinder their confidence and willingness to pursue ARTs (31). The reporting of ARTs stock outs in Guinea because of the ARV stockpile fire at Guinea's central pharmacy (on June 17th, 2017) might have led to HIV patients’ perception of uncertainty and greater risk, thus changing their attitude to ART drug intake.

### Financial burden borne by patients

Although ARTs are provided at no costs in Guinean public health facilities, our results showed that HIV patients are still exposed to financial hardship due to additional healthcare expenditures such as transportation, biomedical exams, costs of opportunistic diseases, and informal payments often made as a gift to health providers. In our study, some patients borrowed money from their relatives or sold their assets such as shoes and mobile phones to cover their care costs. These findings are not specific to Guinea in sub-Saharan African region. Studies in Nigeria and South Africa have reported an increase in HIV patients’ spending on accommodation, special foods and medicines purchased on the days when they visit health facilities (32, 33). Moreover, because of the time spent in health care facilities, HIV patients may incur a loss of income or salary (33). In chronic conditions such as HIV, these costs push thousands of patients into extreme poverty and food insecurity which lead them to stop ARTs (34, 35). There is evidence that bringing HIV care services closer to populations that need them is crucial for achieving global on-going strategies (1). Conversely, our study revealed that HIV patients prefer attending health facilities located far from their residence because of the fear of confidentiality breach.

### The role of psychosocial counsellors

Our study shows the important role that psychosocial counsellors play in relieving health providers of psychosocial support. Furthermore, health managers reported greater patient adherence to health services than before the involvement of psychosocial counsellors in their facilities. Other studies in Kenya and Uganda confirmed these findings and emphasized the positive effects of psychosocial support in optimizing patients’ adherence to care and ARTs (36, 37). A recent literature review in sub-Saharan Africa has also reported that non-professional health workers living with HIV increased the likelihood of HIV patients to adhere to care and ARTs (37). One possible explanation for these findings might be that non-professional health workers spend more time with PLHIV and this enables them to provide excellent education on HIV and medication use (38). In addition, they have the ability to provide social and emotional support to HIV patients (37), especially when they are patients themselves, engaged as peers and sharing their own experience of life and care.

### Practical implications

Our study points to strategies that can be implemented with minimal costs or change to care organization. Firstly, given the importance of a strong mutual trust between patient and provider in ensuring an effective and completed HIV care, it is crucial for providers to restrict access to consultation rooms during HIV patients’ visits, as reported in this study.

Psychosocial counsellors have been identified as key actors in reinforcing and maintaining a confidential relationship with HIV patients and with the provision of psychological support. For Guinea to achieve holistic (social, psychological and physical) care for HIV patients, it is crucial to develop an organizational model of care, which aims to strengthen the role of psychosocial counsellors and to introduce task-shifting between health providers and non-health providers, especially in the case of psychosocial counselling and to consider engaging PLHIV in peer-support.

The essential element of patients’ engagement in care focused on drug provision and the biomedical tests performed. These findings suggest the need to change the mind-set of health providers and patients for the implementation of an effective patient-centred approach in HIV care in Guinea.

### Strengths and limitations of the study

To our knowledge, this is one of the first qualitative studies in Francophone West Africa to conduct an in-depth exploration of the organization and patient-provider's relationship in the context of HIV care. Furthermore, the implementation and reporting of this study also adhered to the internationally recognized Consolidated criteria for reporting qualitative research (COREQ) guidelines (39). However, the study reveals some methodological limitations. We conducted this study in health facilities based in the capital, Conakry. Therefore, the findings may not be representative of the national situation and rural settings. There was a limited number of focus group discussions with psychosocial counsellors. There was a national strike because of fuel price rising which harmed public transportation and thus access of psychosocial counsellors to health facilities. Another limitation was the social desirability bias as our study sites were supported by an international NGO. The strategy used by the research team to limit this bias was to specify our external role, independent from the NGO. The activities of the project were implemented before this study had started.

## Conclusion

This qualitative study provides an in-depth understanding of the organization of health care services, the experience of care for the patients and providers, and the patient-provider relationship in HIV care in Conakry, Guinea. Our findings show a less patriarchal provider-patient relationship with a progressive engagement of HIV patients in their medical care, the importance of reorganizing care pathways, taking in account the role of psychosocial counsellors. There is a need to support the integration of psychosocial counsellor in HIV care provision, as to sustain the benefits of the PACTES project in Guinea.

## Data Availability

The original contributions presented in the study are included in the article/Supplementary Material, further inquiries can be directed to the corresponding author.
